# Telemedicine Use Before and During the COVID-19 Pandemic in People with Alzheimer’s Disease, Multiple Sclerosis, or Parkinson’s Disease: A Cross-Sectional Study Using US Commercial Claims Data

**DOI:** 10.1089/tmr.2024.0030

**Published:** 2024-08-13

**Authors:** Anisha M. Patel, Robert Schuldt, Denise M. Boudreau, Bryan R. Cobb, Nikki Win, Marisa P. McGinley

**Affiliations:** ^1^ Genentech Inc., South San Francisco, California, USA.; ^2^ Cleveland Clinic, Cleveland, Ohio, USA.

**Keywords:** Alzheimer’s disease, multiple sclerosis, Parkinson’s disease, telehealth, telemedicine

## Abstract

**Background:**

During the COVID-19 pandemic, use of telemedicine (TM) increased dramatically, but it is unclear how use varies by characteristics of people with Alzheimer’s disease (AD), multiple sclerosis (MS), or Parkinson’s disease (PD).

**Methods:**

This cross-sectional study used US PharMetrics Plus commercial claims data from January 1, 2019, to December 31, 2021. TM use (≥1 Current Procedural Terminology code) was assessed in each study year (2019, 2020, and 2021) among people with ≥1 inpatient or ≥2 outpatient diagnosis codes ≥30 days apart for AD, MS, or PD. Any TM use and disease-related visits (AD, MS, or PD diagnosis code within TM claim) were summarized, and characteristics of TM users versus nonusers during the pandemic (2020 and 2021) were described.

**Results:**

Among people with AD, MS, or PD, 0.9% used TM in 2019 versus 58.0% in 2020 and 42.5% in 2021. Among TM users in 2020 and 2021, the majority had TM visits related to their neurological disorder (73.2% and 64.6%, respectively). During the pandemic, approximately 25% of total TM visits (*n* = 296,434) were provided by a neurologist. Mean (SD) age of TM users was similar to nonusers (60.5 [15.1] and 61.5 [15.3] years), but TM users were more likely to be female (62% vs 60%), enrolled in Medicare (33% vs 30%), and reside in western (64.2% vs 35.8%) or eastern (61.0% vs 39.0%) regions versus nonusers.

**Conclusions:**

Although results indicate expanded use of and access to TM among people with AD, MS, or PD, differences in patient and health care provider characteristics for TM use were notable.

## Introduction

Neurological disorders are a leading cause of disability and health burden in the United States.^1^ Within the United States, access to specialized neurological care is limited due to a shortage of neurologists and inefficiencies in the health care system, resulting in particularly long wait times to see a specialist.^2–4^ A rising prevalence of neurological disorders,^1^ coupled with limited access to neurologists, has led to a health care access gap.^5^

Telehealth refers to the remote provision of synchronous or asynchronous health care services, education, and monitoring using digital and telecommunication technologies, whereas telemedicine (TM) refers to direct virtual clinical services that occur between a patient and ≥1 provider when participants are separated by distance or time.^2,6–8^ Within the field of neurology, patients with acute stroke have received remote health care services (“telestroke”) for decades.^2,9^ TM has the potential to increase access to health care, but data are limited,^5,10^ and disparities in digital access (known as the “digital divide” [e.g., broadband Internet access]) exist.^11–13^

The COVID-19 pandemic spurred a shift in health care delivery from in-person to virtual or blended care in the United States.^7,14^ Early in the pandemic, TM use expanded rapidly and at peak, the use of TM comprised approximately 50% of overall health care claims.^7^ Among neurologists, >50% used TM at least once during the pandemic.^15^ Although the use of TM among people with neurological disorders has expanded,^5,15^ it is unclear how the use of TM varies in specific populations and what characteristics distinguish TM users from nonusers.

The objective of this study was to evaluate TM use via TM claims for synchronous patient-health care provider visits and access patterns before and during the COVID-19 pandemic among people with Alzheimer’s disease (AD), multiple sclerosis (MS), or Parkinson’s disease (PD). These three neurological disorders were selected because of their high incidence, prevalence, and disease burden.^1^

## Methods

### Data source and study design

This cross-sectional study used IQVIA PharMetrics Plus commercial claims data. PharMetrics Plus contains fully adjudicated medical and pharmacy claims from deidentified patients in all 50 US states and the District of Columbia.^16^ Additional details can be found in IQVIA PharMetrics^®^ Plus fact sheet.^16^ In 2020, the database included approximately 1 million patients covered by Medicare Advantage plans and 7.7 million patients covered by private plans.^17^ The database contains information on patient demographics (e.g., birth year, sex, three-digit zip code, and state of residence); health plan enrollment; and inpatient, outpatient and pharmacy claims.^16^

The study period was January 1, 2019, to December 31, 2021. Data from January 1, 2019, to December 31, 2019, were defined as the “pre-COVID-19” or “prepandemic” period, and data from January 1, 2020, to December 30, 2021, were defined as the “COVID-19” or “pandemic” period. Adult patients (age ≥18 years) were identified if they had ≥11 months of enrollment in medical benefits during the calendar year and ≥1 inpatient or ≥2 outpatient International Statistical Classification of Diseases and Related Health Problems, Tenth Revision, Clinical Modification (ICD-10-CM), codes ≥30 days apart for AD (G30.0, G30.1, G30.8, and G30.9), MS (G35), or PD (G20). TM use, identified using ≥1 Current Procedural Terminology (CPT) code, was assessed in each study year (2019, 2020, and 2021) among identified patients. The following CPT codes were used 99441–99443 for non-face-to-face (i.e., telephone or audio only) TM visits, 99201–99205 for new patients or 99212–99215 for established patients plus modifier codes for real-time, interactive audio/video (GT, GQ, or 95). Any TM use and neurological disorder-related visits (AD, MS, or PD diagnosis code within a TM claim) were summarized for each study year, and characteristics of TM users versus nonusers during the pandemic (2020 and 2021) were examined for each disease area (AD, MS, and PD) and for the total neurological disorder cohort. Use of TM during the pandemic was further examined by patients’ state of residence and by three-digit zip code level.

Comorbidity burdens were defined by the Charlson comorbidity index (CCI), a weighted index used to predict long-term mortality.^18^

### Statistical analysis

Wilcoxon rank sum and Pearson χ^2^ tests were used to test for differences between the characteristics of TM users and nonusers among people with AD, MS, or PD during the COVID-19 pandemic. Uncorrected *P* values are reported. To generate the map of TM use, people were grouped within three-digit zip codes, for which a per-person TM claim rate was calculated and then divided into quintiles based on the per-person TM claim rate. Visualizing the differences in TM use across regions during the study period required calculating the composition of counties based on urban influence codes within each three-digit zip code to illustrate the proportion of counties within each three-digit zip code that were urban, micropolitan/metropolitan, adjacent rural and remote rural counties (Supplementary Table S1). Analyses were performed in RStudio v4.1.2.

### Standard protocol approvals, registrations, and patient consents

The IQVIA database^16^ uses deidentified data and is compliant with the Health Insurance Portability and Accountability Act of 1996. No institutional review board approval was required.

### Data availability

The data that support the findings of this study are available from IQVIA (PharMetrics Plus commercial health claims database). Restrictions apply to the availability of these data, which were used under license for this study.

## Results

### Attrition

Cohorts (2019, 2020, and 2021) and diagnoses (AD, MS, and PD) were not mutually exclusive. For 2019, a total of 101,598 people with AD, MS, or PD were included, of whom 18,539 had AD, 56,579 had MS and 30,797 had PD (Supplementary Table S2). For 2020, a total of 95,715 people with AD, MS, or PD were included, of whom 18,513 had AD, 52,475 had MS, and 29,720 had PD. For 2021, a total of 109,029 people with AD, MS, or PD were included, of whom 20,823 had AD, 57,442 had MS, and 34,598 had PD (Supplementary Table S2).

### TM use before and during the pandemic among people with AD, MS, or PD

Among people with AD, MS, or PD, 0.9% used TM in 2019 compared with 58.0% in 2020 and 42.5% in 2021 (Table 1). Among people with AD, 48.3% used TM in 2020 and 35.7% in 2021; among people with MS, 57.4% used TM in 2020 and 43.3% in 2021; and among people with PD, 61.9% used TM in 2020 and 44.1% in 2021. The mean (SD) number of TM claims among people with any TM visit in 2019 was 1.6 (1.2), in 2020 was 2.9 (2.8), and in 2021 was 2.7 (3.0) (Table 1). A similar mean number of claims was observed for patients in the AD, MS, and PD cohorts during the pandemic (Supplementary Table S3). The mean number of TM claims during the pandemic did not vary by quarter (Supplementary Figure S1).

**Table 1.** TM Visits and Claims Among People with Alzheimer’s Disease, Multiple Sclerosis, or Parkinson’s Disease by Year

	2019^a^ *n* = 101,598	2020^a^ *n* = 95,715	2021^a^ *n* = 109,029
Any TM visit within the year, *n* (%)	933 (0.9)	55,517 (58.0)	46,315 (42.5)
1 TM visit	647 (69.3)	21,134 (38.1)	19,780 (42.7)
>1 TM visit	286 (30.7)	34,383 (61.9)	26,535 (57.3)
Number of TM claims per person among people with any TM visit, mean (SD)	1.6 (1.2)	2.9 (2.8)	2.7 (3.0)
AD/MS/PD-related TM visits among people with any TM visit, *n* (%)	198 (21.2)	40,624 (73.2)	29,915 (64.6)
Visit type, *n* (%)^a^			
≥1 Audio visit	529 (56.7)	19,347 (34.8)	12,405 (26.8)
≥1 Interactive audio/video visit	409 (43.8)	46,217 (83.2)	39,730 (85.8)
Patient type, *n* (%)^b^			
≥1 TM visit as a new patient	71 (7.6)	2148 (3.9)	2038 (4.4)
≥1 TM visit as an established patient	346 (37.1)	45,097 (81.2)	38,591 (83.3)
Percentage of all claims for TM visits within the year	0.2	5.0	3.2
Percentage of TM claims that were AD/MS/PD related within the year	18.3	48.2	43.8

^a^ Not mutually exclusive.

^b^ Unknown patient type not shown.

AD, Alzheimer’s disease; MS, multiple sclerosis; PD, Parkinson’s disease; TM, telemedicine.

Among people with any TM visit, the percentage of disease-related claims for the condition of interest (i.e., AD, MS, or PD) was 21.2% in 2019, 73.2% in 2020, and 64.6% in 2021 (Table 1). Among the disease-specific cohorts during the pandemic, approximately half to three-quarters of all TM visits were for the condition of interest for people with AD (56.2%, 2020; 48.4%, 2021), MS (77.4%, 2020; 70.2%, 2021) and PD (71.6%, 2020; 61.0%, 2021) (Supplementary Table S3).

Prepandemic, the percentage of people using TM who had ≥1 audio visit was similar to the percentage of people who had ≥1 interactive audio/video visit (56.7% and 43.8%, respectively) (Table 1). During the pandemic, however, a higher percentage of TM users had ≥1 interactive audio/video visit than audio visits alone (83.3% vs 34.9%, respectively, in 2020 and 85.8% vs 26.8%, respectively, in 2021), and from 2019 to 2020, the use of interactive audio/video visits nearly doubled (43.8% to 83.2%) and continued to increase in 2021 (85.8%). A similar trend was observed in each disease-specific cohort (Supplementary Table S3).

Both before and during the pandemic, a higher percentage of established patients had TM visits than new patients in the total AD/MS/PD cohort (37.1% vs 7.6%, respectively, in 2019; 81.2% vs 3.9%, respectively, in 2020; 83.3% vs 4.4%, respectively, in 2021) (Table 1). During the pandemic, a high percentage of claims was also observed for established patients among people with AD (73.7%, 2020; 75.3%, 2021), MS (84.6%, 2020; 86.6%, 2021), and PD (78.5%, 2020; 83.3%, 2021) (Supplementary Table S3).

### Demographics and characteristics of TM users during the pandemic

**FIG. 1.**
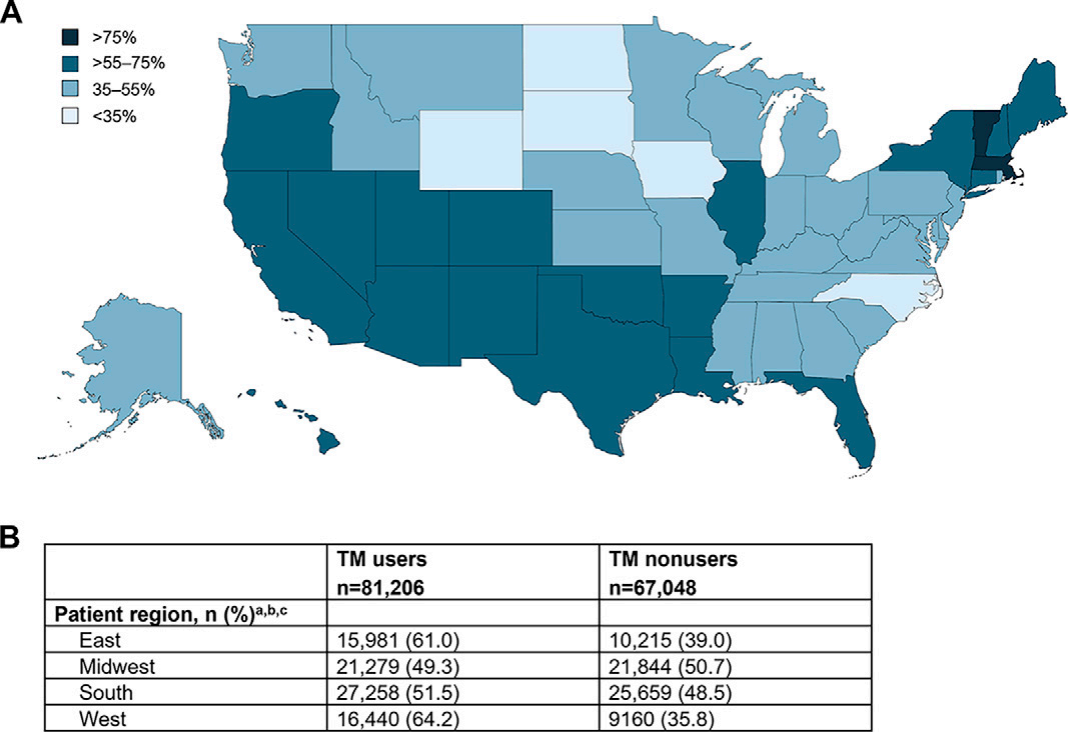
Percentage of TM use by state of residence and region among people with Alzheimer’s disease, multiple sclerosis, or Parkinson’s disease during the pandemic (2020–2021)^a. a^Unknown not shown. ^b^*P* ≤ 0.001. ^c^Row percentages shown. TM, telemedicine.

The mean (SD) age of TM users (*n* = 81,206) with AD, MS, or PD was similar to that of nonusers (*n* = 67,048) (60.5 [15.1] vs 61.5 [15.3] years; *P* < 0.001), and comorbidity burden was similar as well, as shown by the mean (SD) CCI score (1.39 [1.98] vs 1.38 [1.96]; *P =* 0.7). However, TM users were more likely to be female (62.2% vs 59.8%; *P* < 0.001), enrolled in Medicare (33.0% vs 30.5%; *P* < 0.001) followed by commercial insurance (29.7% vs 27.4%; *P* < 0.001), reside in western (64.2% vs 35.8%; *P* < 0.001), and eastern (61.0% vs 39.0%; *P* < 0.001) regions, and were less likely to be self-insured (36.8% vs 41.6%; *P* < 0.001) compared with nonusers (Table 2; Fig. 1). In addtion, TM users had a higher mean (SD) number of health care claims during the pandemic than nonusers (41.9 [58.2] vs 31.8 [54.2]; *P* < 0.001) (Table 2).

**Table 2.** Characteristics of TM Users Versus TM Nonusers Among People with Alzheimer’s Disease, Multiple Sclerosis, or Parkinson’s Disease During the Pandemic (2020–2021)

	TM users *n* = 81,206	TM nonusers *n* = 67,048
Age, mean (SD), years^a^	60.5 (15.1)	61.5 (15.3)
Female, *n* (%)^a^	50,478 (62.2)	40,126 (59.8)
Product type, *n* (%)^a^		
HMO	14,636 (18.0)	11,778 (17.6)
PPO	58,861 (72.5)	47,307 (70.6)
Other^b^	7709 (9.5)	7963 (11.9)
Payer, *n* (%)^a^		
Commercial	24,145 (29.7)	18,366 (27.4)
Medicaid	260 (0.3)	313 (0.5)
Medicare	26,830 (33.0)	20,448 (30.5)
Self-insured	29,888 (36.8)	27,859 (41.6)
Other^c^	83 (0.1)	62 (0.1)
CCI score, mean (SD)^d^	1.39 (1.98)	1.38 (1.96)
CCI score, *n* (%)^a^		
0	40,333 (49.7)	33,677 (50.2)
1	8688 (10.7)	5378 (8.0)
2+	32,185 (39.6)	27,993 (41.8)
Total claims, mean (SD)^a^		
Any claim	41.9 (58.2)	31.8 (54.2)
Any ND-related claim	12.5 (26.1)	9.8 (26.6)
Neurological disorder, *n* (%)^a^		
AD	12,933 (15.9)	17,056 (25.4)
MS	41,683 (51.3)	31,583 (47.1)
PD	26,590 (32.7)	18,409 (27.5)

^a^ *P* ≤ 0.001.

^b^ Defined as indemnity, point of service, consumer direct, health savings account, or unknown.

^c^ Defined as unknown or State Children’s Health Insurance Program.

^d^ *P* < 0.7.

AD, Alzheimer’s disease; CCI, Charlson comorbidity index; HMO, health maintenance organization; MS, multiple sclerosis; ND, neurological disorder; PD, Parkinson’s disease; PPO, preferred provider organization; TM, telemedicine.

People with MS or PD were more likely to be TM users than nonusers (51.3% vs 47.1% and 32.7% vs 27.5%, respectively), whereas people with AD were less likely to be TM users than nonusers (15.9% vs 25.4%) (*P* < 0.001 for all; Table 2). TM users with MS or PD were also more likely to be female than nonusers (77.8% vs 73.5% and 38.7% vs 35.0%, respectively; *P* < 0.001) (Supplementary Table S4). Among people with AD or PD, TM users had a higher mean (SD) CCI score than nonusers (AD, 4 [2] vs 3 [2]; PD, 2 [2] vs 1 [2]) (Supplementary Table S4). In all disease-specific cohorts (AD, MS, and PD), TM users were more likely to be enrolled in Medicare and less likely to be self-insured than nonusers (Supplementary Table S4). Aligning with the overall AD/MS/PD population, TM users with MS or PD were much more likely to live in western and eastern regions, and TM users with AD were more likely to live in western regions (Supplementary Table S5).

### Provider specialty during the pandemic

**FIG. 2.**
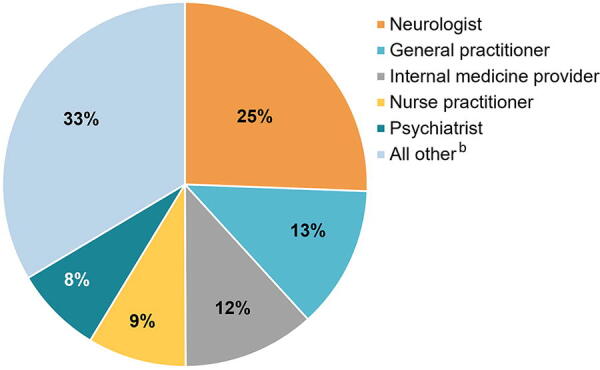
Provider specialty for TM visits^a^ for people with Alzheimer’s disease, multiple sclerosis, or Parkinson’s disease during the pandemic (2020–2021). ^a^N = 296,434. ^b^Includes 58 provider specialties and excludes other facilities or unknown. The top three provider specialties in the “all other” category for the neurological disorder cohort were physician assistant (3.2%), cardiologist (2.4%), and anesthesiologist (1.5%). TM, telemedicine.

Among people with AD, MS, or PD, 25.6% of total TM visits (*n* = 296,434) during the pandemic were provided by a neurologist, followed by general practitioner (12.6%), internal medicine provider (11.7%), nurse practitioner (8.7%), and psychiatrist (7.8%) (Fig. 2). All other provider specialties comprised 33.6% of total TM visits; the top three specialties in this category were physician assistant (3.2%), cardiologist (2.4%), and anesthesiologist (1.5%). A similar pattern of results was observed for TM visits among people with MS and PD, but a neurologist was less likely to provide TM visits to people with AD (Supplementary Figure S2).

### TM use by state of residence and zip code

The percentage of people with AD, MS, or PD using TM was highest in Massachusetts (81.3%), Vermont (77.1%), California (72.4%), Hawaii (69.9%), and New Hampshire (67.4%) and lowest in North Dakota (6.7%), Iowa (19.0%), and Wyoming (26.5%) (Supplementary Table S6). A similar pattern of results was observed for each specific neurological disorder (i.e., AD, MS, and PD) (Supplementary Table S6).

**FIG. 3.**
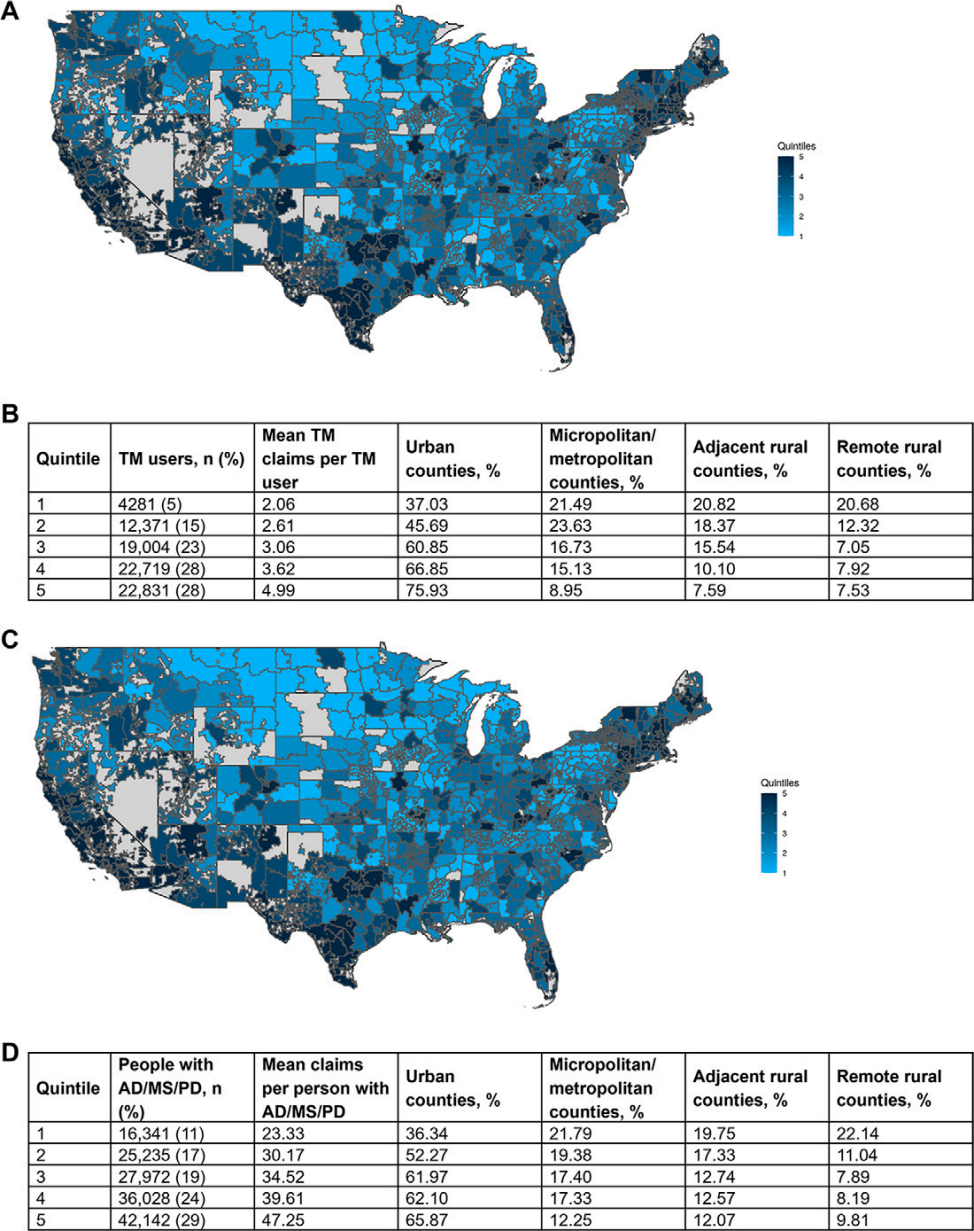
Quintiles of **(A,B)** TM use and **(C,D)** claims^a^ by 3-digit zip code during the pandemic (2020–2021). ^a^Alaska and Hawaii not shown. Pt, patient; TM, telemedicine.

Across all quintiles, TM users were more likely to reside in three-digit zip codes primarily composed of urban counties (75.93% of counties in quintile 5 vs 37.03% of counties in quintile 1) and less likely to reside in remote rural counties (7.53% of counties in quintile 5 vs 20.68% of counties in quintile 1) (Fig. 3A,B). The use of TM was also higher in coastal regions in New England, California, and parts of Texas (Fig. 3A). While the overall pattern of TM use showed higher per-patient claim counts in three-digit zip codes primarily composed of urban counties (65.81% of counties in quintile 5 vs 36.34% of counties in quintile 1) and lower per-patient claim counts in three-digit zip codes primarily composed of remote rural counties (9.81% of counties in quintile 5 vs 22.14% of counties in quintile 1), the concentration of the highest utilization zip codes centered around the midwestern region (Fig. 3C,D).

## Discussion

The COVID-19 pandemic led to the rapid adoption of telehealth.^7,14^ Results from this study demonstrated expanded use of and access to TM among people with neurological disorders, AD, MS, and PD, during the COVID-19 pandemic; however, we found disparities in TM use by sex, geographic region, insurance type, neurological disorder, and comorbidity burden. Importantly, differences in demographics and geographic regions between TM users and nonusers did not appear to correlate with incidence and prevalence of neurological disorders and may reflect potential access gaps.

Consistent with studies of other diseases, a dramatic increase in TM visits was observed during the COVID-19 pandemic in our study among people with any neurological disorder.^7,14,15,19^ Approximately half of people with any neurological disorder in a commercially insured population used TM during the pandemic. TM users were more likely to be female than male in the overall cohort consistent with results from studies published before the pandemic.^20,21^ While women are 3 times more likely to have MS than men,^21^ men are 1.5 times more likely to have PD than women,^22^ which may suggest a preference for TM among women with PD, independent of incidence/prevalence. It is unclear if TM use among women with MS is because of higher incidence/prevalence of MS among women.

TM users were more likely to be enrolled in Medicare followed by a commercial plan and less likely to be self-insured. These results may reflect policy changes that occurred at the beginning of the pandemic that allowed for coverage of in-home TM for Medicare beneficiaries.^23^ However, coverage of in-home TM use by Medicare will likely expire December 31, 2024.^23,24^ This is concerning because older individuals who receive Medicare face additional barriers to accessing health care that TM could help address.^25^ Although mean CCI scores were not higher for TM users versus non TM users in the overall cohort, TM users with AD or PD had slightly but significantly higher mean CCI scores than nonusers. Across AD, MS, or PD, many TM visits involved an established patient visit. Previous research has found that an existing patient–provider relationship may encourage TM use.^20^

Consistent with observations in other studies,^19^ a majority of TM visits during the pandemic used interactive audio/video components. The sustained increase in interactive audio/video components of TM visits from 2020 to 2021 underscores the need for reliable broadband Internet access for long-term utility of TM to avoid perpetuation of a digital divide that excludes historically marginalized communities.^26,27^ Results from this study found comparatively low use of TM in three-digit zip codes primarily composed of remote rural counties (Fig. 3). Hispanic/Latino patients are less likely to choose video visits than White patients, and Black/African American patients are more likely to choose both audio-only and audio/video visits than patients of any other race or ethnicity.^20^

Only one-quarter of TM visits among the overall AD/MS/PD cohort were provided by a neurologist, although 65% to 73% of TM visits were related to the condition of interest, based on provider specialty information available in the database. Among people with AD, only 14% of TM visits were provided by a neurologist. Globally, access to specialized neurological care is poor or limited due to a shortage of neurologists and inefficiencies in the health care system.^2–4^ TM could help to maintain access, improve disparities (e.g., lack of transportation), and provide continuity of care for people with neurological disorders; however, additional research is needed to understand how TM can help optimize delivery of multidisciplinary care for these patients across different providers and to understand the proportion of individuals with AD, MS, or PD who never met with a neurologist during a TM visit.

TM users were more likely to reside in eastern and western regions of the United States. Among the overall AD/MS/PD cohort, TM users were most likely to reside in Massachusetts, Vermont, and California and least likely to reside in North Dakota, Iowa, and Wyoming. Several reasons may explain the high use of TM in Massachusetts, Vermont, and California, including more expansive TM policies^28^ or residing patient or provider preferences. Of states with low TM use, North Dakota, Iowa and Wyoming have been identified as “neurology deserts” based on projected disparities between patient demand and provider supply.^3,29^ The use of TM was higher in urban counties than remote rural counties (Supplementary Table S4), consistent with concerns of a digital divide in TM access. In general, lower TM use does not correlate with lower incidence/prevalence of neurological disorders, which suggests that geographic differences identified in this study may reflect true access gaps.^1^

The current, claims-based, real-world findings in neurology patients build on inequities observed in TM use during the pandemic among general patient populations in smaller cohort and cross-sectional studies.^30–32^ These studies were geographically restricted to the Midwest^30,32^ and Northeast^31^ and consistently exposed lower odds of TM use for non-White race, noncommercially insured, and older patients with lower socioeconomic status. To our knowledge, our study is the first to have assessed telehealth utilization at the national level across different neurology patient cohorts. Our study adds evidence that although TM use increased globally during the pandemic, certain groups continued to receive inequitable care across neurological disorders (i.e., AD, MS, and PD) based on their personal or geographic characteristics across the United States.

There are several limitations and biases inherent to the use of claims datasets. The dataset included participants who were predominantly enrolled in commercial or Medicare Advantage insurance, so the results may not generalize to individuals who are uninsured or are living in rural, underserved areas. Southern and midwestern regions were slightly overrepresented in the dataset. The claims data did not include socioeconomic characteristics (e.g., area deprivation index, race and ethnicity, and education) that can influence access to TM, and no information on disease severity was available, which precluded an assessment of TM use by participants’ disease severity. Data are collected for the purpose of insurance reimbursement, and values for nonessential fields such as provider specialty may be incomplete or missing. In addition, provider specialty may reflect the facility at which services were received rather than the specialty of the care provider. Finally, the correlation between TM use and the incidence and prevalence of neurological disorders was not formally explored in this study; although, overall, lower TM use was not found to correlate with reported incidence and prevalence^1^ of neurological disorders assessed.

In summary, this study found disparities in TM use by sex, geographic region, insurance type, neurological disorder, and comorbidity burden. Although TM use has the potential to expand access to health care for people with neurological disorders and reduce inefficiencies to optimize health care, infrastructure and supportive policies are essential. Further analyses are needed to inform gaps in the accessibility of TM as well as its long-term utility and effect on health outcomes in people with neurological disorders, particularly after policy changes that may occur following the COVID-19 pandemic.

## Abbreviations Used

AD: Alzheimer’s disease
CCI: Charlson Comorbidity Index
CPT: Current Procedural Terminology
ICD-10-CM: International Statistical Classification of Diseases and Related Health Problems Tenth Revision Clinical Modification
MS: Multiple sclerosis
PD: Parkinson’s disease
TM: Telemedicine
