# Supplementary material for: Telemedicine Use Before and During the COVID-19 Pandemic in People with Alzheimer’s Disease, Multiple Sclerosis, or Parkinson’s Disease: A Cross-Sectional Study Using US Commercial Claims Data
Source: Telemed Rep. 2024 Aug 13;5(1):247–55. doi: 10.1089/tmr.2024.0030 (PMC11342049; doi:10.1089/tmr.2024.0030)
Supplement: Supplementary Figure S1 [file tmr.2024.0030_supplement_data.pdf]

SUPPLEMENTAL MATERIAL

eTable 1. Zip-Code County Composition Definitions (Baer et al., 1997)

Term	Definition
Urban	Metropolitan areas with <1 (small-in) or >1 million (large-in) residents
Micropolitan/metropolitan	Areas adjacent large or small metropolitan area or not adjacent to a metropolitan area
Adjacent rural	Adjacent to a large metropolitan area, or to a small metropolitan area with town of ≥2500 residents or adjacent to a small metropolitan area and not containing a town of ≥2500 residents
Remote rural	Areas adjacent to micropolitan area and containing a town of 2500–19,999 residents or adjacent to a micropolitan area and not containing a town of ≥2500 residents or not adjacent to a metropolitan or micropolitan area and containing a town of ≥2500 residents or not adjacent to a metropolitan or micropolitan area and not containing a town of ≥2500 residents

Confidential | Not for distribution

eTable 2. Attrition

	AD	MS	PD	AD, MS and/or PD
**2019**				
Meets study criteria[a]	43,153	71,249	47,803	160,271
Age ≥18 years	19,294	70,664	38,491	127,356
≥11 Months of enrollment	18,539	56,579	30,797	101,598
**2020**				
Meets study criteria[a]	39,049	66,023	45,241	148,623
Age ≥18 years	19,325	65,608	37,857	121,732
≥11 Months of enrollment	18,513	52,475	29,720	95,715
**2021**				
Meets study criteria[a]	39,049	67,869	47,114	152,504
Age ≥18 years	21,959	67,554	40,931	129,381
≥11 Months of enrollment	20,823	57,442	34,598	109,029

AD, Alzheimer's disease; MS, multiple sclerosis; PD, Parkinson's disease.
[a]Study criteria included ≥1 inpatient or ≥2 outpatient International Statistical Classification of Diseases and Related Health Problems, Tenth Revision, Clinical Modification (ICD-10-CM), codes ≥30 days apart for AD (G30.0, G30.1, G30.8, G30.9), MS (G35) or PD (G20).

Confidential | Not for distribution

eTable 3. TM Visits and Claims Among People With Alzheimer's Disease, Multiple Sclerosis or Parkinson's Disease by Year[a]

	2020			2021		
	**AD n=18,513**	**MS n=52,475**	**PD n=29,720**	**AD n=20,823**	**MS n=57,442**	**PD n=34,598**
**Any TM visit, n (%)**	8948 (48.3)	30,113 (57.4)	18,399 (61.9)	7436 (35.7)	24,895 (43.3)	15,265 (44.1)
1 TM visit	3551 (39.7)	12,068 (40.1)	6331 (34.4)	3297 (44.3)	10,808 (43.4)	6253 (41.0)
>1 TM visit	5397 (60.3)	18,045 (59.9)	12,068 (65.6)	4139 (55.7)	14,087 (56.6)	9012 (59.0)
Number of TM claims per person among people with any TM visit, mean (SD)	2.8 (2.6)	2.7 (2.7)	3.1 (3.1)	2.7 (2.7)	2.6 (2.8)	2.9 (3.3)
TM visits related to condition of interest among people with any TM visit, n (%)	5026 (56.2)	23,315 (77.4)	13,173 (71.6)	3600 (48.4)	17,485 (70.2)	9313 (61.0)
**Visit type, n (%)[b]**						
≥1 Audio visit	4157 (46.5)	8298 (27.6)	7780 (42.3)	2817 (37.9)	5153 (20.7)	4943 (32.4)
≥1 Interactive audio/video visit	6759 (75.5)	26,081 (86.6)	14,828 (80.6)	5784 (77.8)	22,175 (89.1)	12,751 (83.5)
**Patient type, n (%)[c]**						
≥1 TM visit as a new patient	279 (3.1)	1219 (4.0)	728 (4.0)	311 (4.2)	1138 (4.6)	594 (3.9)
≥1 TM visit as an established patient	6597 (73.7)	25,469 (84.6)	14,449 (78.5)	5596 (75.3)	21,551 (86.6)	12,395 (81.2)
**Percentage of all claims for TM visits**	3.0	5.9	17.9	2.0	4.0	11.5
**Percentage of TM claims for condition of interest**	35.5	52.7	42.3	32.9	48.6	40.3

AD, Alzheimer's disease; MS, multiple sclerosis; PD, Parkinson's disease; TM, telemedicine.

[a]For 2019, too few data to report for the disease-specific cohorts.

[b]Not mutually exclusive.

[c]Unknown patient type not shown.

eTable 4. Characteristics of TM Users vs TM Nonusers Among People With Alzheimer's Disease, Multiple Sclerosis or Parkinson's Disease During the Pandemic (2020-2021)

	AD		MS		PD	
	**TM users n=12,933**	**TM nonusers n=17,056**	**TM users n=41,683**	**TM nonusers n=31,583**	**TM users n=26,590**	**TM nonusers n=18,409**
**Age, mean (SD), years**[a]	75 (8)	76 (7)	51 (12)	50 (12)	69 (10)	68 (10)
**Female, n (%)**[b]	7743 (60)	10,485 (61)	32,442 (78)	23,198 (73)	10,293 (39)	6443 (35)
**Product type, n (%)**[c]						
HMO	3252 (25)	4741 (28)	6711 (16)	3723 (12)	4673 (18)	3314 (18)
PPO	8316 (64)	10,169 (60)	31,233 (75)	24,372 (77)	19,312 (73)	12,766 (69)
Other[d]	1365 (11)	2146 (13)	3739 (9)	3488 (11)	2605 (9.8)	2329 (13)
**Payer, n (%)**[c]						
Commercial	1767 (14)	2002 (12)	16,171 (39)	11,754 (37)	6207 (23)	4610 (25)
Medicaid	43 (0.3)	149 (0.9)	168 (0.4)	108 (0.3)	49 (0.2)	56 (0.3)
Medicare	8758 (68)	11,033 (65)	5315 (13)	2647 (8.4)	12,757 (48)	6768 (37)
Self-insured	2340 (18)	3846 (23)	19,992 (48)	17,048 (54)	7556 (28)	6965 (38)
Other[e]	25 (0.2)	26 (0.2)	37 (<0.1)	26 (<0.1)	21 (<0.1)	10 (<0.1)
**CCI score, mean (SD)**[c]	4 (2)	3 (2)	1 (1)	1 (1)	2 (2)	1 (2)
**CCI score, n (%)**[f]						
0	0 (0)	0 (0)	28,330 (68.0)	23,753 (75.2)	12,003 (45.1)	9924 (53.9)
1	0 (0)	0 (0)	5215 (12.5)	3128 (9.9)	3473 (13.1)	2250 (12.2)
2+	12,933 (100)	17,056 (100)	8138 (19.5)	4702 (14.9)	11,114 (41.8)	6235 (33.9)

AD, Alzheimer's disease; CCI, Charlson Comorbidity Index; HMO, health maintenance organization; MS, multiple sclerosis; PD, Parkinson's disease; PPO, preferred provider organization; TM, telemedicine.

[a]For MS, *P*=0.003; for AD and PD, *P*<0.001.

[b]For AD, *P*=0.005; for MS and PD, *P*<0.001.

[c]*P*<0.001.

[d]Defined as indemnity, point of service, consumer direct, health savings account or unknown.

[e]Defined as unknown or State Children's Health Insurance Program.

[f]Alzheimer's disease has a minimum CCI score of 2 (Quan et al., 2005; Quan et al., 2011)

Confidential | Not for distribution

eTable 5. TM Use by Region During the Pandemic (2020-2021)

	AD		MS		PD	
	**TM users n=12,933**	**TM nonusers n=17,056**	**TM users n=41,683**	**TM nonusers n=31,583**	**TM users n=26,590**	**TM nonusers n=18,409**
**Patient region, n (%)**[a,b]						
East	2217 (50.5)	2172 (49.5)	8957 (62.4)	5389 (37.6)	4807 (64.4)	2654 (35.6)
Midwest	3241 (36.4)	5655 (63.6)	11,626 (52.5)	10,512 (47.5)	6412 (53.0)	5677 (47.0)
South	3244 (35.1)	5998 (64.9)	14,990 (54.7)	12,392 (45.3)	8254 (54.0)	7269 (46.0)
West	3661 (53.8)	3148 (46.2)	6054 (65.0)	3258 (35.0)	6725 (70.9)	2754 (29.1)

AD, Alzheimer's disease; MS, multiple sclerosis; PD, Parkinson's disease; TM, telemedicine.
[a]*P*<0.001.
[b]Unknown not shown.

Confidential | Not for distribution

eTable 6. Percentage of TM Use by State of Residence and Neurological Disorder During the Pandemic (2020-2021)

State	AD, MS and/or PD	AD	MS	PD
MA	81.3	83.2	82.6	66.3
VT	77.1	73.6	81.6	64.1
CA	72.4	78.9	78.7	62.6
HI	69.9	71.3	76.5	68.6
NH	67.4	74.1	74.4	58.3
NJ	65.9	70.0	71.8	51.5
TX	64.8	64.3	69.8	48.8
NM	64.5	67.2	73.1	45.3
CT	64.2	72.9	75.8	53.4
AZ	61.7	65.1	67.6	42.7
OK	61.4	60.7	65.7	45.9
IL	60.3	61.1	62.7	47.5
UT	59.7	52.6	63.9	45.5
CO	59.6	65.6	66.4	40.9
FL	58.8	59.1	63.3	46.1
ME	57.6	62.8	65.0	44.7
OR	57.0	66.8	72.5	42.9
NV	56.8	65.4	60.7	47.6
LA	56.7	54.9	63.1	36.3
AR	56.1	66.0	62.1	35.1
NY	55.5	60.8	58.5	46.9
GA	54.3	59.3	63.8	41.8
DC	52.7	55.1	50.8	55.0
WA	52.7	53.5	55.5	44.5
SC	52.5	51.7	56.2	36.7
MI	51.4	50.5	52.8	36.8
OH	50.6	58.8	62.0	39.3
PA	49.5	49.7	50.4	39.8
RI	49.1	45.7	52.1	33.7
IN	48.2	54.5	57.5	35.9
VA	47.9	55.2	52.8	40.3
ID	47.7	53.8	47.8	37.5
MS	46.6	51.7	46.8	39.7
MD	46.5	50.6	48.3	35.8
DE	46.5	48.8	47.7	35.3
MT	45.7	46.6	47.5	26.5

KY	45.2	53.1	53.8	34.8
NE	44.2	36.8	49.2	17.8
WI	44.0	48.1	49.9	28.5
AL	42.6	45.8	44.5	26.1
KS	42.4	44.1	44.1	24.6
MO	40.3	45.1	47.8	27.9
MN	37.7	48.6	35.3	34.3
TN	36.5	34.7	40.6	20.9
AK	36.4	38.8	35.1	45.5
WV	36.1	46.3	33.5	27.2
NC	32.9	31.8	34.2	32.0
SD	27.0	28.6	28.7	6.3
WY	26.5	26.3	26.3	24.0
IA	19.0	23.5	17.3	17.1
ND	6.7	7.3	6.4	11.5
Unknown	57.7	68.9	63.6	45.8

AD, Alzheimer’s disease; MS, multiple sclerosis; ND, neurological disorder; PD, Parkinson’s disease; TM, telemedicine.

Confidential | Not for distribution

eFigure 1. Mean Number of Quarterly **(A)** Healthcare Claims Among People With Alzheimer's Disease, Multiple Sclerosis or Parkinson's Disease and **(B)** TM Claims Among TM Users With Alzheimer's Disease, Multiple Sclerosis or Parkinson's Disease During the Pandemic (2020-2021)

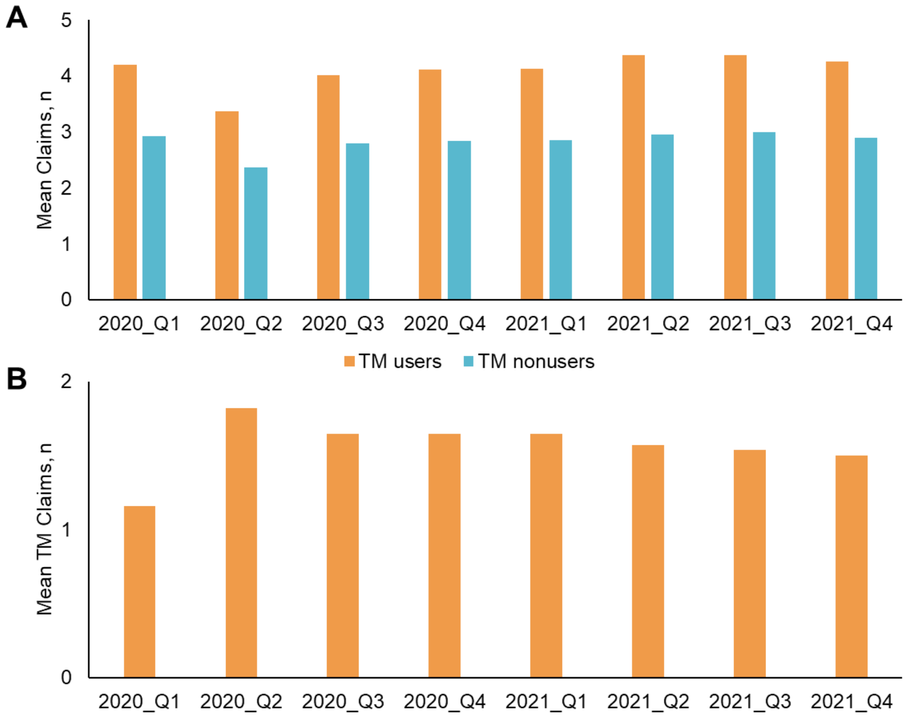

Q, quarter; TM, telemedicine.

Confidential | Not for distribution

eFigure 2. Provider Specialty for TM Visits[a] for People With (A) Alzheimer's Disease, (B) Multiple Sclerosis or (C) Parkinson's Disease During the Pandemic (2020-2021)

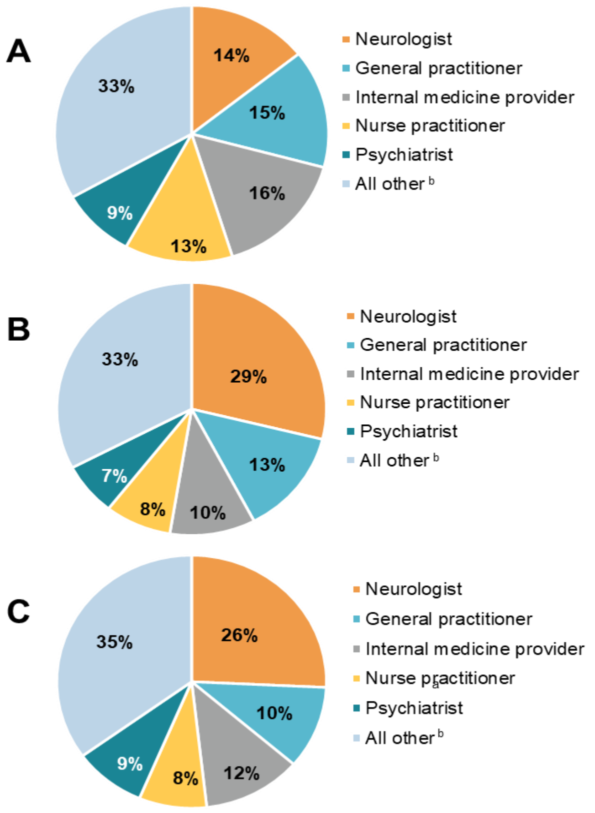

AD, Alzheimer's disease; MS, multiple sclerosis; PD, Parkinson's disease; TM, telemedicine.
[a]AD, n=44,303; MS, n=145,514; PD, n=106,617.
[b]Includes 58 provider specialties and excludes other facility or unknown. The top 3 provider specialties in the "all other" category for the AD cohort were cardiologist (3.3%), physician assistant (2.6%) and geriatrician (2.3%); for the MS cohort were physician assistant (3.8%), anesthesiologist (2.4) and durable medical equipment provider/home healthcare practitioner (2.0%); and for the PD cohort were cardiologist (3.5%), physician assistant (2.6%) and gastroenterologist (2.2%).

Supplemental References

Baer LD, Johnson-Webb KD and Gesler WM. What is rural? A focus on urban influence codes. J Rural Health. 1997;13(4):329–333. doi.org/10.1111/j.1748-0361.1997.tb00975.x

Quan H, Li B, Couris CM, et al. Updating and validating the Charlson comorbidity index and score for risk adjustment in hospital discharge abstracts using data from 6 countries. *Am J Epidemiol.* 2011;173(6):676-682. doi:10.1093/aje/kwq433

Quan H, Sundararajan V, Halfon P, et al. Coding algorithms for defining comorbidities in ICD-9-CM and ICD-10 administrative data. *Med Care.* 2005;43(11):1130-1139. doi:10.1097/01.mlr.0000182534.19832.83

Confidential | Not for distribution
